# Amphetamine actions rely on the availability of phosphatidylinositol-4,5-bisphosphate

**DOI:** 10.1186/1471-2210-11-S2-A19

**Published:** 2011-09-05

**Authors:** Florian Buchmayer, Klaus Schicker, Gerald Stübiger, Peter J Hamilton, Petra Geier, Andreas Jurik, René Weissensteiner, Thomas Steinkellner, Heinrich J Matthies, Therese Montgomery, Marie-Therese Winkler, Jae-Won Yang, Marion Holy, Gerhard F Ecker, Aurelio Galli, Valery Bochkov, Stefan Boehm, Harald H Sitte

**Affiliations:** 1 Institute of Pharmacology, Center of Physiology and Pharmacology, Medical University of Vienna, 1090 Vienna, Austria; 2 Department of Molecular Physiology and Biophysics, Vanderbilt University, Nashville, TN 37232, USA; 3 Department of Medicinal Chemistry, University of Vienna, 1090 Vienna, Austria

## Background

Neuronal functions, such as excitability or endo- and exocytosis, require phosphatidylinositol-4,5-bisphosphate (PIP_2_) since ion channels and other proteins involved in these processes are regulated by PIP_2_. Monoamine transporters control neurotransmission by removing monoamines from the extracellular space. They also display channel properties, but their regulation by PIP_2_ has not been reported. The psychostimulant amphetamine acts on monoamine transporters to stimulate transportermediated currents and efflux and thereby increases the levels of extracellular monoamines.

## Methods and results

Direct or receptor-mediated activation of phospholipase C (PLC) reduced membrane PIP_2_ and amphetamine-evoked currents through recombinant serotonin transporters; extracellular application of a PIP_2_-scavenging peptide mimicked this effect. PLC activation also diminished amphetamine-induced reverse transport without altering transmitter uptake. Inhibition of reverse transport by PLC activation was also observed in brain slices and with recombinant dopamine and noradrenaline, but not GABA transporters; rises in intracellular Ca^2+^ or activation of protein kinase C were not involved in these effects.

## Conclusions

These data demonstrate for the first time PIP_2_ dependence of reverse transport and current in monoamine transporters.

